# Multipotent Mesenchymal Stromal Stem Cell Expansion by Plating Whole Bone Marrow at a Low Cellular Density: A More Advantageous Method for Clinical Use

**DOI:** 10.1155/2012/920581

**Published:** 2011-10-15

**Authors:** Katia Mareschi, Deborah Rustichelli, Roberto Calabrese, Monica Gunetti, Fiorella Sanavio, Sara Castiglia, Alessandra Risso, Ivana Ferrero, Corrado Tarella, Franca Fagioli

**Affiliations:** ^1^Stem Cell Transplantation and Cellular Therapy Unit, Pediatric Onco-Hematology Department, Regina Margherita Children's Hospital, 10126 Turin, Italy; ^2^Department of Pediatrics, University of Turin, Piazza Polonia 94, 10126 Turin, Italy; ^3^Molecular Biotechnology Center, University of Turin, P.za Polonia 94, 10126 Turin, Italy

## Abstract

Mesenchymal stem cells (MSCs) are a promising source for cell therapy due to their pluripotency and immunomodulant proprieties. As the identification of “optimal” conditions is important to identify a standard procedure for clinical use. Percoll, Ficoll and whole bone marrow directly plated were tested from the same sample as separation methods. The cells were seeded at the following densities: 100 000, 10 000, 1000, 100, 10 cells/cm^2^. After reaching confluence, the cells were detached, pooled and re-plated at 1000, 500, 100, and 10 cells/cm^2^. Statistical analyses were performed. Cumulative Population Doublings (PD) did not show significant differences for the separation methods and seeding densities but only for the plating density. Some small quantity samples plated in T25 flasks at plating densities of 10 and 100 cells/cm^2^ did not produce any expansion. However, directly plated whole bone marrow resulted in a more advantageous method in terms of CFU-F number, cellular growth and minimal manipulation. No differences were observed in terms of gross morphology, differentiation potential or immunophenotype. These data suggest that plating whole bone marrow at a low cellular density may represent a good procedure for MSC expansion for clinical use.

## 1. Introduction

In recent years, a large number of studies have shown that mesenchymal stem cells (MSCs) represent an attractive option for new therapeutic approaches, due to their plasticity and differentiative potential. MSCs are multipotent stem cells that are able to differentiate into different lineages including mesodermal, ectodermal, and endodermal type cells [1–4]. MSCs can be easily isolated by their ability to adhere to plastic generating single-cell-derived colonies [5, 6] that can be expanded to obtain high numbers of cells for clinical use in cell and gene therapy for a number of human diseases [1]. 

Several methods have been described for isolating MSCs from bone marrow (BM), including the use of immuno-magnetic beads, density gradient separation, and direct BM plating [2, 7–11]. Density gradients such as Ficoll or Percoll centrifugation are commonly used to isolate MSCs from human BM [2, 8, 11, 12] whereas direct plating is commonly used for cells from rats [10], mice [13], and rabbits [14] which have limited available BM. Hemopoietic contamination, due to the presence of macrophages, endothelial cells, and lymphocytes which also adhere to plastic, is often present in the early BM monolayer [2, 15]. However, only fibroblast-like spindle-shaped cells proliferate and form colonies termed colony forming unit-fibroblasts (CFU-Fs) which are representative of the more highly proliferative cells in MSCs [6, 16]. On the basis of isolation and expansion protocols, the CFU-Fs originate MSCs with different proliferative and differentiative potentials which may be either subtle or significant [9, 17]. The International Society for Cellular Therapy proposed three minimal criteria to identify MSCs (an abbreviation used to indicate *multipotent mesenchymal stromal cell*): (1) the adherence to plastic; (2) the specific surface antigen expression (positivity for CD105, CD73, CD90 and the lack of expression of CD45, CD34, CD14 or CD11b, CD79a or CD19 and HLA class II; (3) the multipotent capacity to differentiate into osteoblasts, adipocytes, and chondrocytes under standard *in vitro* differentiating conditions [18].

The safety, feasibility, and efficiency of MSC transplantation for clinical use are currently the object of studies, and, as several protocols use extremely high numbers of MSC (until 10^9^), the identification of “optimal” conditions for *in vitro* cell culture should also be investigated.

Isolation methods, including medium, plastic, seeding density, growth factors, and chemicals, influence the expansion, differentiation, and immunogenic properties of MSCs. Futhermore, donor age and disease stage [19, 20] can also influence MSC yield, proliferation rate, and differentiation potential. BM MSCs are usually isolated from BM mononuclear cells obtained after gradient separation and for their capacity to adhere to plastic.

Percoll, a suspension of colloidal silica particles, widely used at different densities to separate cells, organelles, viruses and other subcellular particles, or Ficoll a polymer of sucrose, traditionally used to separate mononuclear cells and lymphocytes, have both been used at densities of 1.073 g/mL [2, 21–23] and 1.077 g/mL [24–26], respectively, to isolate MSCs with high proliferative and differentiative potential.

In this study, using the same BM sample, we isolated MSCs from healthy donors using different separation and expansion methods. We used Ficoll, Percoll, and direct BM plating as the separating methods and tested different seeding and plating cellular densities to verify the best method to obtain a high number of MSCs for clinical use. 

## 2. Material and Methods

### 2.1. Harvest and Preparation of MSCs

Bone marrow (BM) cells were harvested from the iliac crest of adult or pediatric Caucasian donors who underwent bone marrow collection for a related patient after informed consent. When available, we also used an unfiltered bone marrow collection bag (Baxter Healthcare Corporation, IL, USA) which was normally discarded before the BM infusion. The bag was washed 3 times with Phosphate Buffer Saline (PBS) 1X (Lonza, Verviers, Belgium), and the cells were collected at 200 g for 10 minutes. An aliquot of whole BM was counted and plated directly in MSC Medium (Lonza, Verviers, Belgium) containing 10% Foetal Bovine Serum (FBS) at the various densities in T25 o T75 flasks (Becton Dickinson, Franklin Lakes, NJ, USA). The remaining part of the BM sample was divided into 2 parts for the Percoll and Ficoll gradient separation. The cells were layered on a Percoll (Sigma Aldrich, St. Louis, MO, USA) gradient (1.073 g/mL density) according to a previously reported method [20] and on a Ficoll (Biochrom, Milton Road, Cambridge, UK) gradient (1.077 g/mL density). The cells were centrifuged at 1100 g for 30 minutes, and 400 g for 30 minutes respectively. The cells in the interphase were recuperated, washed twice with PBS 1X (200 g for 10 minutes), seeded in a MSC Medium containing 10% FBS and maintained at 37°C with an atmosphere of 5% CO_2_ at the following densities: 100 000, 10 000, 1000, 100, and 10 cells/cm^2^. After 5 days, the nonadherent cells were removed and refed every 3-4 days and when they reached confluence, they were detached, pooled, and replated for a further 3–5 passages at 1000, 500, 100, and 10 cells/cm^2^.

### 2.2. MSC Analysis

The cells were counted and analyzed at each passage for cellular growth, viability and immunophenotype by cytofluorimetric analysis.

### 2.3. MSC Clonogenic and Proliferation Potential

The clonogenic potential of isolated MSCs from the 3 different BM fractions (whole BM, mononuclear cell [MNC] fraction after Ficoll and Percoll gradient) were tested by fibroblastic-colony-forming unit (CFU-F) assay. The cells were seeded at the different densities, and the medium changed every 3-4 days. MSC clonogenic precursors (CFU-F) were scored macroscopically after 2 weeks, and clusters of more than 50 cells were considered colonies. All the experiments were performed in duplicate.

On average, the CFU-Fs were counted by 2 different operators. The CFU-Fs were indicated as the fibroblastic clones obtained from the starting cellular compartment of the whole BM.

The cellular expansion growth rate of MSCs was evaluated by cell count in a Burker Chamber at each passage and expressed in terms of population doubling (PD) using the formula log⁡*N*/log⁡2, where *N* is the cell number of the confluent monolayer divided by the initial number of cells seeded [20].

### 2.4. Cytofluorimetric Analysis of MSCs

The identification of adherent cells was performed by flow cytometry analysis. At each passage, 200 000–500 000 cells were stained for 20 minutes with anti CD105 PE (Immunostep S.L, Salamanca, Spain), CD45 FITC, CD14 PE, CD73 PE, CD44 PE, CD29 FITC (Becton Dickinson, San Jose, CA, USA), CD105 PE, CD166 FITC, CD90 FITC, CD106 PE (Beckman Coulter, Brea, CA, USA.). The labeled cells were thoroughly washed with PBS 1X and analyzed on a FACScanto II (Becton Dickinson) with the DIVA software program. The percentage of positive cells was calculated using the cells stained with Ig FITC/PE as a negative control.

### 2.5. Differentiation Potential Assay

For differentiation experiments, from the 1st to the 5th passages MSCs were cultured in osteogenic, adipogenic and chondrogenic medium (Lonza) according to the manufacturer's instructions. Briefly, 20 000 and 50 000 cells were plated in a T-25 flask for osteogenesis and adipogenic culture conditions, respectively, allowing the cells to adhere to the culture surface for 24 hours in MSC medium (Lonza). To induce osteogenesis and adipogenesis, the medium was replaced with specific complete induction medium (Lonza). After 21 days, osteogenic differentiation was demonstrated by the accumulation of calcium (crystalline hydroxyapatite detection by Von Kossa staining) in separated cells plated in chamber slides in the same culture conditions.

For the adipogenic differentiation, adipogenic induction and maintenance medium were alternatively used every 3-4 days, and the presence of intracellular lipid vesicles visible after 2-3 weeks' culture was assessed by Oil Red O staining.

For chondrogenic differentiation, an aliquot of 250 000 cells was washed twice with incomplete chondrogenic medium (Lonza) in 15 mL polypropylene culture tubes. Finally, the cells were resuspended in complete chondrogenic medium, centrifuged and, without aspirating thesurnatant, the tubes were incubated at 37°C in a humidified atmosphere of 5% CO_2_. Chondrogenic differentiation was due to the growth of the cells as cellular aggregates floating freely in suspension culture with Transforming Growth Factor (TGF)-beta3. The pellet was included in paraffin and stained with Alcian Blue to identify the presence of hyaluronic acid and sialomucin.

### 2.6. Telomere Length (TL) Evaluation

TL evaluation was carried out by Southern Blot (SB) analysis as described elsewhere [27]. Briefly, 22 *μ*g of DNA were digested by mixing HinfI (20 U) and RsaI (20 U) (Roche Diagnostic, Mannheim, Germany) and incubating at 37 for 2 h (Figure 5). Resulting DNA fragments were then separated on a 0.8% agarose gel by electrophoresis in 1X TAE running buffer and 5 *μ*L of ethidium bromide. Separated DNA was subsequently transferred to a positively charged nylon membrane (RocheDiagnostic Mannheim, Germany). After an overnight transfer, in order to fix DNA fragments the membrane was exposed to UV light for 10 minutes. Hybridization was carried out with the TeloTAGG Telomere Length Assay Kit (Roche Diagnostics, Manheim, Germany).

Membranes were submerged in a prehybridization solution and then incubated in the hybridization solution (2 *μ*L of the digoxigenin (DIG)-labeled telomere-specific probe added to the prehybridization solution) for 3 h at 62°C. Then, membranes with DNA fragments linked to telomere probes were incubated with a digoxigenin-specific antibody covalently coupled to alkaline phosphatase (AP).

The results were visualized using AP metabolizing CDP-Star, a highly sensitive chemiluminescent substrate.

The light signal was recorded on X-ray film (Lumi-Film Chemiluminescent Detection Film, Roche Diagnostic, Mannheim, Germany) and scanned for analysis.

Median TR length was calculated using the software quantity One by Biorad (Hemel Hempstead, UK).

### 2.7. Statistical Analysis

Cell growth data were analyzed by SPSS 15 for Windows (SPSS Inc, Chicago, IL). The results were expressed as medians and ranges. The differences between paired samples were evaluated by Friedman's test [28, 29] and a post hoc multiple comparison analysis using the Least Significant Difference (LSD) method.

All statistical tests were two-sided and significant for a *P* value <0.05.

## 3. Results

### 3.1. MSC Harvest and Preparation

Ten bone marrow samples were collected from donors: 3 over 18 years of age (age range: 39–50 years) (2 male and 1 female) and 7 (all male) with ages younger than 18 years (age range: 0.5–10 years). The study was conducted according to the Helsinki Declaration. The whole BM was counted and seeded for all the experiments; the remaining part of the sample was separated into equal fractions for MSC separation by Ficoll and Percoll, respectively. The median of initial cell numbers for gradient separation was 69 × 10^6^ (range: 21–82 × 10^6^) and, after separation, the total recovery corresponded to 13.5% and 15.7%, respectively after Ficoll and Percoll (median value; range Ficoll: 5.0–18.9%, Percoll: 1.0–28.8%). These data showed that there were no differences between Ficoll and Percoll in terms of cell count and recovery after isolation.

### 3.2. MSC Isolation

Adherent cells were observed in all the samples after 3–5 days' culture and in the following 2 weeks an adherent monolayer was achieved. The BM cells rapidly generated a confluent layer of cells with an elongated, fibroblastic shape. The viability at each passage was always over 98%. No morphological differences were observed on the MSCs isolated from whole BM, Ficoll and Percoll, but when early passage cells were compared with late passage cells, MSCs showed a different morphology. The cells increased in size and showed a polygonal morphology with evident filaments in the cytoplasm especially when isolated from the adult donors.

### 3.3. MSC Analysis

**Figure 1:**
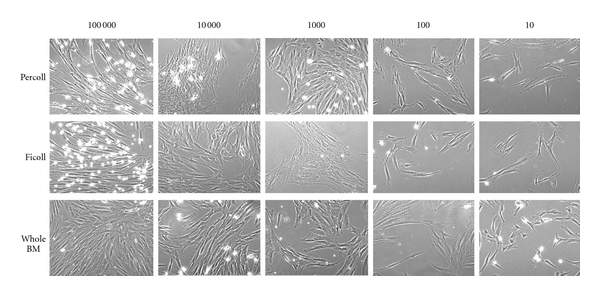
Phenotype of different clones observed after Percoll and Ficoll gradient separation and whole BM 10 days from seeding at different densities.

MSCs isolated from healthy donors were analyzed for the first 3 passages with a median interval between one passage and the next of 16 days (range: 7–40). We observed heterogeneous MSC preparation and a distinct population of spindle-shaped or flat MSCs in the flasks, although no morphological differences were observed in the 3 preparations (Ficoll, Percoll, and whole BM).  Figure 1 shows the clones from different methods after 10 days from seeding at the various densities. In 7 out of 10 cultures we did not observe clones in culture seedings at 10 and 100 cells. Interestingly, we observed clones at lower densities only in the 3 samples obtained from the washout of discarded BM collection bags and filters. In addition, these samples were obtained from child patients.

**Figure 2:**
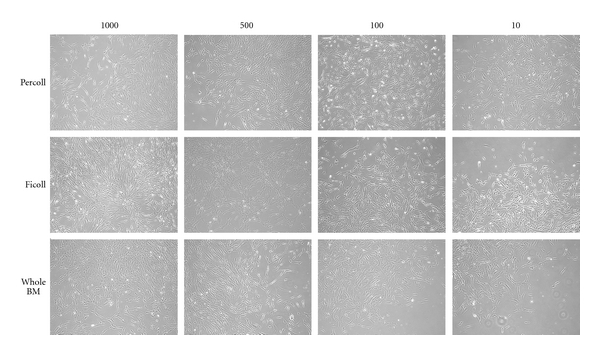
Phenotype of different clones observed after Percoll and Ficoll gradient separation and whole BM 7 days from plating.

After detachment, the cells replated at 1000, 500, 100, and 10 cells/cm^2^ formed clones that reached confluence in a median of 16 days (range: 7–40). The cells pooled and replated at the second and third passages also formed clones in all preparations.  Figure 2 shows the clones at the second passage after 7 days from plating.

In order to compare the effect of 3 different separation methods and different densities on the proliferative capacity of MSCs, 3 BM samples were plated to ascertain the CFU-F frequency. The CFU-F number was calculated in relation to the initial cell number in the BM sample and by comparing the 3 separation methods. The results were as follows: whole BM showed a median of 104.4 CFU-F (range: 7.2–179.7) per 10^6^ cells; after Ficoll and Percoll separation, BM showed a median of 3.8 (range: 2.7–49.8) and 0.1 (range: 0–16.4) CFU-F per 10^6^. We observed no direct correlation between the number of cells plated and the number of CFU-Fs counted when analyzing the effect of the densities. In particular, at seedings of 10, 100, 1000, 10 000, and 100 000 cells/cm^2^, we observed a median of 0 (range: 0-0), 0 (range: 0.0–44.4); 6.2 (range: 0.7–104.0); 62.5 (7.4–111.7) and 11.3 (3.1–13.8), respectively. Significant differences were noted among seedings at 10 with 10 000 and 100 000 cells/cm^2^ between 100 with 10 000 cells/cm^2^  and 1 000 with 10 000. The analysis of the 3 different separation methods and the different seeding densities, considering the grand total of all values obtained for whole BM, Ficoll and Percoll (*N* = 10), as shown in Table 1(a), and the grand total of all values of each density, as shown in Table 1(b), did not show significantly different growth rate values at the first passage (*P* = 0.49 and *P* = 0.51, resp.).

On 4 samples, it was possible to perform a complete analysis for cumulative PD from the 1st to the 3rd passages, and the results showed that BM always showed a more advantageous growth compared to Ficoll and Percoll separation even though the statistic analysis showed no significant differences (*P* = 0.653; *P* = 0.931 and *P* = 0.528 at the 1st, 2nd and 3rd passages, resp.).

As we did not observe MSC clones in more than half of the primary cultures for the seeding at 10 and 100 cells/cm^2^, these densities were excluded for the final analysis. At the 1st passage, we observed a significant statistical difference between the seedings at 1000 and 10 000 (*P* = 0.028), but not between 10 000 and 100 000 cells/cm^2^. 

The analysis on the effect of plating density at 10, 100, 500, and 1000 cells/cm^2^  at the 1st, 2nd, and 3rd passages is summarized in Table 3. In particular, we observed significant statistical differences at the 2nd passage between plating at 10 compared to 500 and 1000 cells/cm^2^ at both the 2nd and 3rd passages (*P* = 0.001 and *P* = 0.017).

### 3.4. Viability Evaluation

Trypan blue staining analysis showed a viability of between 98%–100% in all the analyzed samples with no differences between the two groups. The same results were confirmed after 7AAD staining in the cytofluorimetric analysis.

### 3.5. Immunophenotype Analysis by Flow Cytometry

**Figure 3:**
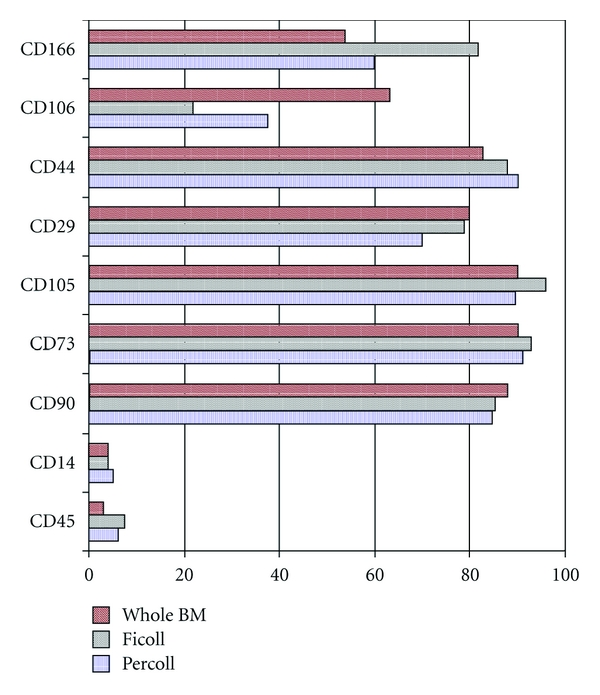
Immunophenotype analysis of MSCs isolated after Percoll and Ficoll gradient separation and whole BM at the 1st passage.

During the 1st 3 passages, the cells were analyzed at each passage for the expression of CD45 and CD14, haematopoietic surface antigens; CD90; CD29, CD44; CD105; CD166 and CD106, CD73. At the 1st passage, MSCs isolated from whole BM were CD45, CD14 negative with an antigen expression less than 5% (the median was 3.0% with a range of 0.0–6.5% and 3.5% with a range of 0.0–7.0%, resp.), while they showed a high expression of CD90 (median of 90.0%, range: 65.0–93.5%), CD29 (median of 78%, range: 61.0–97.0%), CD44 (median of 83.0%, range: 65.0–99.0%) and CD105 (median of 90.0%, range: 65.0–95.0%) and a lower expression of CD106, CD166 adhesion molecules (median of 63% with a range of 2.4–88.0% and 54% with a range of 53.0–96.0%, resp.). In the MSC isolated from Ficoll and Percoll, at the 1st passage the immunophenotype showed a weak hematopoietic contamination because the median expression of CD45 was 7.5% (range: 0.0–48.0%) and 6.0% (range: 1.0–44%:), respectively, and because the expression for CD14 was 5.0% (range: 0.0–35%) and 4.0% (range: 0.0–24%), respectively, for the Ficoll and Percoll separation methods. High levels of CD90, CD29, CD44, and CD105 with values over 80% and variable percentages of CD106 and CD166 were observed without significant differences even in the cells separated by Ficoll and Percoll. In Figure 3, the median antigen expression which was analyzed at the 1st passage on the cells isolated by 3 different methods (a pool of the different seeding densities) is represented as a histogram. During the expansion time, the MSCs were negative for the hemopoietic antigen, whereas at each passage they expressed high percentages of CD90, CD73, CD29, CD44, and CD105 positive cells with the median antigen expression being over 80%.

### 3.6. Differentiation Potential Assay

**Figure 4:**
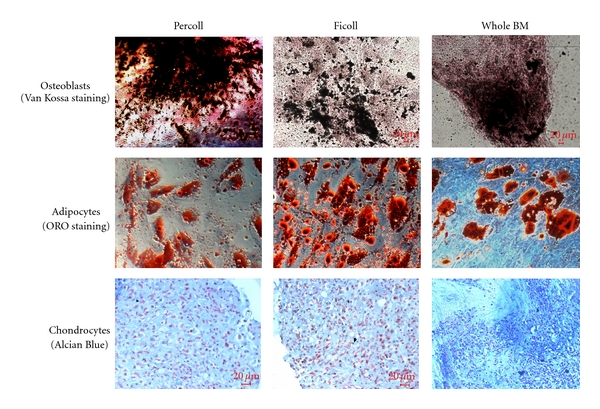
Differentiation of MSCs isolated after Percoll and Ficoll gradient separation and whole BM at the 3rd passage.

**Figure 5:**
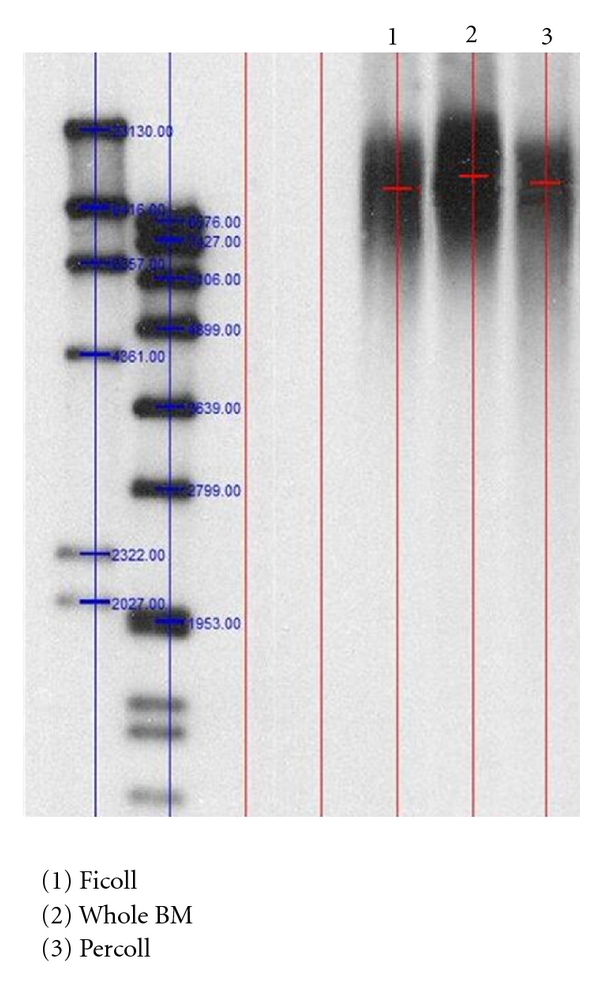
Telomere Restriction Fragment (TRF) measurement by Southern Blot analysis in one representative experiment on cells isolated after Percoll and Ficoll gradient separation and whole BM at the 3rd passage.

All samples induced into differentiation with specific medium showed a multi-potential capacity because all the MSCs, independently of the separation methods and seeding and plating densities, differentiated into osteoblasts, adipocytes, and chondrocytes as shown in Figure 4. In 2 cultures of MSCs isolated from whole BM at the 1st passage, we observed the spontaneous presence of some clones of adipogenic and osteoblastic cells, respectively (data not shown).

### 3.7. Telomere Length Analysis

We analyzed 9 samples harvested from pediatric healthy donors of BM (male and female); the MSCs used for this analysis were all at the 3rd seeding passage and were obtained by Ficoll (*N* = 3), Percoll (*N* = 3), and direct whole BM plating (*N* = 3).

We observed a median of 11543 pb (range: 12181–11504 pb), 12906 (range: 13406–12016), and 10725 pb (range: 12060–10578 pb), respectively, for the percoll, whole BM, and Ficoll group.

## 4. Discussion

In recent years, a number of insights into MSC biology, as well as their immune regulatory proprieties and regenerative potential, have provided support for considering MSCs as a good candidate for cellular therapy for regenerative medicine, cancer gene therapy, and the treatment of immunologic diseases.

Although the culture of MSCs has been studied for over 30 years, standard criteria to isolate and characterize these multipotent stem cells have yet to be developed.

Several methods have been described to enrich BM MSCs for clinical applications. In this study, we tested, from the same BM sample, 3 different isolation methods and several different seeding and plating cellular densities. We analyzed the cellular growth, the number of CFU-Fs, the immunophenotype and differentiative potential in all isolated cultures to ascertain the optimal culture condition to isolate and expand MSCs for clinical applications.

MSCs isolated by their adherence to plastic culture surfaces have characteristic properties that have been well defined by a number of investigators [6, 30]. It is therefore difficult to compare data from different laboratories for both the different isolation and expansion methods and for the high variability of the cells inside the culture. It is often possible to note that in the cultures two morphologically distinct cells are present [6, 30, 31]: Type I cells that are spindle shaped and grow rapidly, and Type II cells that are broad and grow slowly. Moreover, in our experiments, we observed that the greater the number of passages, the higher the increase in Type II cell numbers. We also observed cells with intermediate morphologies. Other authors also demonstrated that samples of human MSCs obtained from iliac crest aspirates varied widely in their expandability in culture [32]. The variation was not explained by either the gender or the age of the donors, nor was it explained by the number of nucleated cells in the sample, but apparently reflected a sampling variation in marrow aspirates from the iliac crest, since the variation was seen between two samples taken from the same volunteer at the same time [32]. MSCs arise from the complex architectural structures of perivascular cells that incompletely separate the marrow from capillaries [30], and the yield of MSCs apparently varies with the presence of such architectural structures in the aspirate site. 

We observed no significant differences for the separation methods because MSCs isolated from Percoll, Ficoll, or whole BM showed significant differences in terms of morphology, growth rate at the 1st passage, cumulative PD, immunophenotype and differentiative potential. The descriptive analysis, however, showed major cellular growth in terms of absolute values and a minor hematopoietic contamination at the 1st passage with whole BM rather than with Percoll or Ficoll.

As the expandability of the MSC culture might be predictable, not from the initial growth rates in the 1st or 2nd passages, but on the basis of the assays for CFUs [6, 25, 33], we performed the CFU-F assays to compare the effect of 3 different separation methods in different cellular seeding densities. Considering the initial cell number in the BM sample, we obtained a much higher number of CFU-F in whole BM conditions rather than after gradient separation.

Moreover, it was interesting to note that the telomere length on MSCs isolated from whole BM was longer than MSCs isolated from Percoll and Ficoll methods. Thus, by cultivating the cells from whole BM we probably isolated more immature MSCs. This latter aspect is worthy of further research.

Even though MSC isolation and expansion by GMP manufactured gradient media have recently been reported [34], this study confirmed that directly plated BM offers a more advantageous method in terms of CFU-F number, minimal manipulation, hematopoietic contamination, and cellular growth (descriptive analysis). Our results (as has also recently been described by Capelli et al. [35] and Lucchini et al. [36]) show that whole BM separation methods represent a good procedure for MSC expansion for clinical use compared to MSCs obtained by gradient separation. To standardize a method of isolation and expansion, the most suitable cellular condition should be used for all samples. We therefore excluded seeding at 10 and 100 cells/cm^2^, since we observed MSC clones in less than half of the primary cultures, whereas, at the 1st passage, we observed a significant statistical difference between the seedings at 1000 and 10 000, but not between 10 000 and 100 000 cells/cm^2^. The CFU-F count was significantly higher at 10 000 cells/cm^2^, therefore the use of this seeding density might prove to be the most advantageous condition. At this seeding density, a 10 mL BM sample, which contains approximately 100 million WBC, would require 10 000 cm^2^ or 16 T630 cm^2^ Cell Factories (flasks used for large scale cell culture). The time to confluence of these cultures would be 2 to 6 weeks which would require only media changes and might offer, at the first passage, the availability of cellular products (about 160 million cells) for clinical use. The procedure proposed would provide a high number of cells starting from a small quantity of BM. A patient would therefore only undergo a BM biopsy and not an invasive procedure such as BM collection. 

The results on cellular growth in terms of cumulative PD confirmed other authors' data that a low plating density results in higher yields and a faster expansion of MSCs [6, 19, 24, 33, 37]. We observed that small spindle-shaped cells in some cultures grew more rapidly at a low plating density. If however, MSCs isolated at the 1st passage were mostly broad and uneven, the expansion was slower and the cells were senescent at a low plating density. An other aspect of MSC *in vitro *aging was observed when the cells were plated at lower densities (10,100) after 4-5 passages (data not shown).

Moreover, we observed clones at lower densities only in the 3 samples obtained from the washout of discarded bone marrow collection bags and filters. These results confirmed those explained by Capelli et al., that is, filtration results in preferential trapping in the filters of hMSC precursors with good proliferative potential, with the consequent enrichment of these cells in the washouts compared with BM.

In conclusion, we observed that, in agreement with other groups with wide experience in this field, [33, 38–40], MSC populations have a diverse repertoire of distinct subpopulations, whose proliferative, immunological, and biological proprieties remain indeterminate. Phinney [40] demonstrated the biochemical heterogeneity of these subpopulations, rather than their stem-like character, contribute more significantly to the therapeutic potential of MSCs.

To our knowledge, this is the first comparative study of different isolation and expansion methods with or without gradient separation. Therefore, the plating of whole BM at a low cellular density may represent a more advantageous procedure for MSC expansion for clinical use compared to MSCs obtained by gradient separation.

## Figures and Tables

**Table:** (a) Analysis of different density effects on seeding (first passage).

*N* = 10			Whole BM	Ficoll	Percoll

Median			8.56	1.76	.69
Minim			.12	.16	.00
Maxim			419.23	111.18	433.26

**Table:** (b) Analysis of different density effects on seeding (first passage).

*N* = 10	10 cells/cm^2^	100 cells/cm^2^	1000 cells/cm^2^	10 000 cells/cm^2^	100 000 cells/cm^2^

Median	.00	2.33	2.66	1.64	.41
Minim	.00	.00	.00	.20	.14
Maxim	531.34	205.20	29.12	5.96	5.52

**Table 2:** Analyses of separation method effects on seeding in terms of cumulative PD.

1st passage			
*N* = 4 (*P* = 0.653)	Whole BM	Ficoll	Percoll
Median	3.11	3.12	2.62
Minim	1.99	.56	1.92
Maxim	5.53	3.89	3.84

2nd passage			
*N* = 4 (*P* = 0.931)	Whole BM	Ficoll	Percoll

Median	4.55	2.68	5.88
Minim	11.41	7.62	8.00
Maxim	6.67	7.16	6.47

3rd passage			
*N* = 4 (*P* = 0.528)	Whole BM	Ficoll	Percoll

Median	10.13	10.50	8.81
Minim	8.60	5.07	8.03
Maxim	15.66	11.11	12.14

**Table 3:** Analyses of seeding density effects in terms of cumulative PD.

1st passage				
*N* = 4 (*P* = 0.068)	10 cells/cm^2^	100 cells/cm^2^	500 cells/cm^2^	1000 cells/cm^2^
Median	5.28	3.27	2.08	2.28
Minim	2.49	.68	.58	1.39
Maxim	5.57	4.65	3.07	2.63

2nd passage				
*N* = 4 (*P*=0.001)*	10 cells/cm^2^	100 cells/cm^2^	500 cells/cm^2^	1000 cells/cm^2^

Median	10.45	6.82	4.48	4.48
Minim	8.33	4.76	2.90	2.88
Maxim	12.06	10.15	6.99	5.94

3rd passage				
*N* = 4 (*P*=0.017)*	10 cells/cm^2^	100 cells/cm^2^	500 cells/cm^2^	1000 cells/cm^2^

Median	14.85	9.89	8.14	6.37
Minim	13.41	6.78	3.82	4.92
Maxim	18.78	13.69	9.84	8.52
